# Clinicopathological characteristics and features of molecular subtypes of breast cancer at high altitudes

**DOI:** 10.3389/fonc.2022.1050481

**Published:** 2023-01-13

**Authors:** Qi Chen, Cheng-Bin Duan, Ye Huang, Kun Liu

**Affiliations:** Department of Medical Oncology, Hospital of Chengdu Office of People’s Government of Tibetan Autonomous Region (Hospital. C. T.), Chengdu, China

**Keywords:** molecular subtype, high altitude, pathology, breast cancer, tibet

## Abstract

**Background:**

Breast cancer is one of the major malignancies threatening women’s health worldwide. The incidence of breast cancer at high altitudes increased over the years. But few studies focused on the characteristics of clinicopathology and molecular subtypes among breast cancer at high altitudes, which are still unknown. Tibet, with an average altitude over 4000 meters, is a representative city at high altitudes, lying in the Qinghai-Tibetan Plateau in southwestern China. This study aimed to identify the clinicopathological characteristics and features of molecular subtypes among Tibetan women with breast cancer, and provide evidence for cancer prevention and personalized therapeutics in high-altitude regions.

**Methods:**

Between May 2013 and March 2022, 104 Tibetan women from high-altitude regions (Tibetan-group) and 34 Han Chinese women from low-altitude regions (Han-group), consecutively diagnosed with breast cancer in the Hospital of Chengdu Office of People’s Government of Tibetan Autonomous Region, were included in the study. We retrospectively reviewed the clinical character, altitudes of residence, tumor size, lymph nodes metastasis, distant metastasis, pathological type, immunohistochemical index, and molecular subtype.

**Results:**

In the study, we calculated the patient delay, equal to the period from symptoms onset to hospital visits. The patient delay of Tibetan-group was 7.47 ± 11.53 months, which was significantly longer than that of Han-group, 7.22 ± 22.96 months (p<0.05). Body Mass Index (BMI) was significantly different (p<0.05). Tumors in Tibetan-group were significantly larger than those in Han-group, 4.13 ± 2.98cm and 2.51 ± 0.82cm in diameter, respectively (p<0.05). According to ordinal logistic regression analysis, exposure to high altitudes might result in more advanced T stage (OR=2.45 95%CI 1.10-5.44). 41.3% (43/104) of cases in Tibetan-group had lymph node positive disease, whereas the percentage was found in 38.26% (13/34) in Han-group(p<0.05). The distribution of molecular subtypes was quite significantly different between two groups (p<0.05), according to the comparison of constituent ratios.

**Conclusion:**

Our study verified that breast cancer at high altitudes possessed its own unique clinicopathological characteristics and distinct features of molecular subtypes. It broadened the understanding of this heterogenous disease and also provided valuable evidence for cancer prevention and personalized therapeutics of breast cancer at high altitudes.

## 1 Introduction

Breast cancer is one of the major malignancies, posing a great threat to women’s health worldwide. According to statistics, female breast cancer had become the most commonly diagnosed cancer, with an estimated 2.26 million new cases in 2020, surpassing lung cancer as the first of cancer incidence (1). Similarly, the incidence of breast cancer at high altitudes increased over the years (2). But few studies focused on the characteristics of clinicopathology and molecular subtypes among breast cancer patients at high altitudes, which are still unknown. Tibet, with an average altitude over 4000 meters, is a representative city at high altitudes, lying in the Qinghai-Tibetan Plateau in southwestern China. Due to its unique climate, geographical location, ethnicity, lifestyle, religion, and economy, breast cancer patients in Tibet may present special disease features. Nowadays, researchers have realized that breast cancer is not a single disease, but a heterogenous complex disease containing several subtypes (3–5). Since the molecular intrinsic subtypes were first presented, they provided various important information to study the heterogeneity of breast cancer, leading into a new era of classified therapy in breast cancer. This study aimed to identify the clinicopathological characteristics and features of molecular subtypes among Tibetan women with breast cancer, and provide evidence for cancer prevention and personalized therapeutics in high-altitude regions.

## 2 Materials and methods

### 2.1 Patients

The study retrospectively reviewed the women who were consecutively diagnosed with breast cancer in the Hospital of Chengdu Office of People’s Government of Tibetan Autonomous Region between May 2013 and March 2022. All patients underwent surgical resection or ultrasound-guided core needle biopsy. The diagnosis was confirmed by histopathology or cytopathology. Patients diagnosed pathologically as primary invasive breast cancer were eligible for inclusion. We defined the patients at high altitudes as those who were Tibetan residents, living permanently at altitudes > 2500 m since they were born. And we defined the patients at low altitudes as those who were Han Chinese, living permanently at altitudes ≤ 1000 m since they were born. Patients who migrated from high altitudes to low altitudes or migrated from low altitudes to high altitudes were excluded. Other exclusion criteria included: parents of patients were migrants, and history of other malignancy. According to the altitude of residence, the patients were divided into the Tibetan-group (high-altitude) and the Han-group (low-altitude). The study was approved by the ethic committee of the Hospital of Chengdu Office of People’s Government of Tibetan Autonomous Region. Because of the retrospective nature of the study, informed consent was waived.

### 2.2 Methods

We retrospectively reviewed the following data: age, the altitude of residence, Body Mass Index (BMI), menstrual status, age at menopause, symptoms, time of symptoms onset, time of hospital visit, imaging tests, tumor size, lymph nodes metastasis, distant metastasis, pathological type, immunohistochemical characteristics, and molecular subtype. We calculated the patient delay, equal to the period from symptoms onset to hospital visits. Tumor size was measured by the largest contiguous dimension of a tumor focus according to pathological criteria among patients who underwent radical surgery. It was according to clinical criteria among patients who didn’t undergo the surgery due to distant metastasis or refusal to surgery. TNM stage was assessed according to the 8th edition of the American Joint Committee on Cancer (AJCC) staging manual.

Pathological evaluation: Estrogen Receptor (ER)/Progesterone Receptor (PR) was defined positive if it was stained in ≥1% of nuclei in tumor cells, and ER/PR was defined negative if it was stained in <1% of nuclei in tumor cells or non-stained (6). Human Epidermal Growth Factor Receptor 2(HER-2), detected by immunohistochemistry (IHC): +++ was positive and + was negative. When HER-2 was scored ++ by IHC, fluorescent *in situ* hybridization (FISH) should be additionally adopted to evaluate the amplification of HER-2 further. If HER-2 amplification occurred, HER-2 (++) was classified as HER-2 positive. If no HER-2 amplification was found, HER-2 (++) was classified as HER-2 negative (7). Ki-67 was evaluated by the percentage of positive invasive tumor cells with any nuclear staining. When the percentage ≥14%, Ki-67 was recorded high and when the percentage <14%, Ki-67 was recorded low (8).

In the study, cases from both groups were classified into four subtypes, including luminal A (ER -and/or PR-positive, HER2-negative, Ki-67 low), luminal B (ER- and/or PR-positive, HER2-negative, Ki-67 high) or (ER- and/or PR-positive, HER2-positive, any Ki-67), HER2-enriched (ER and PR-negative, HER2-positive), and Triple negative (ER and PR negative, HER2-negative), according to St. Gallen consensus criteria (8).

### 2.3 Statistical analysis

Values were compared by the student ‘s t test. Categorical data was compared by Chi-squared test. And when n<5, Fisher’s exact test was performed. Ordinal logistic regression analysis was used to calculate the Odds Ratio (OR). Statistical significance was considered by two-tailed test with p<0.05. The statistical analyses were performed using IBM SPSS statistics 26.0 software.

## 3 Results

### 3.1 Clinical characters

Between May 2013 and March 2022, 138 female patients were enrolled in the study. Among them, 104 patients were Tibetans from high-altitude regions (Tibetan-group), accounting for 75.4%. Their altitudes of residence ranged from 2720 m to 5200 m, and the median altitude was 3650 m. And 34 patients were Han Chinese from low-altitude regions (Han-group), accounting for 24.6%. Their altitudes of residence ranged from 49 m to 1000 m, and the median altitude was 492 m. There was a significant difference in altitude between two groups (p<0.05). Among Tibetan-group, age at diagnosis ranged from 26 to 80 years, and the average age was 47.96 ± 10.56 years, while age at diagnosis among the Han-group ranged from 19 to 70 years, and the average age was 47.96 ± 10.56years (p>0.05). Post-menopausal cases accounted for 41.3% (43/104) in Tibetan-group, and it was found in 47.1% (16/34) in Han-group. The average age at menopause was 48.40 ± 3.47years in Tibetan-group, and it was 50.56 ± 4.68 years in Han-group. No significant difference was observed in terms of menstrual status and menopause age between two groups(p>0.05). In the study, we calculated the patient delay, equal to the period from symptoms onset to hospital visits. Symptoms such as palpable painless breast lump, breast pain, palpable axillary nodes, nipple retraction, nipple discharge, and changes in skin were included. The patient delay of Tibetan-group was 7.47 ± 11.53 months, which was significantly longer than that of Han-group (7.22 ± 22.96 months) (p<0.05). There was a significant difference in Body Mass Index (BMI) between Tibetan-group (26.17 ± 4.80) and Han-group (23.98 ± 3.17) (p<0.05). Tumors in Tibetan-group were significantly larger than those in Han-group, with 4.13 ± 2.98cm and 2.51 ± 0.82cm in diameter, respectively (p<0.05). According to ordinal logistic regression analysis, exposure to high altitudes might result in more advanced T stage (OR=2.45 95%CI 1.10-5.44).

41.3% (43/104) of cases in Tibetan-group had lymph node positive disease, whereas the percentage was found in 38.26% (13/34) in Han-group(p<0.05). 7.69% (8/104) of patients in Tibetan-group had distant metastasis when they were initially diagnosed, whereas the percentage was found in 5.88% (2/34) in Han-group (p>0.05) (**Table 1**).

**Table 1:** Clinical characteristics in Tibetan-group and Han-group.

Clinical character	Tibetan-group	Han-group	p value
Age(years) Altitude of residence(m)	47.96 ± 10.56 3650 ± 488.55	47.96 ± 10.56 492 ± 151.35	0.104 [**0.000**]
Menstrual status [n (%)]			
post-menopausal pre-menopausal	43(41.3%) 61(58.7%)	16(47.1%) 18(52.9%)	0.588
Average age at menopause(years)	48.40 ± 3.47	50.56 ± 4.68	0.058
Patient delay(months)	7.47 ± 11.53	7.22 ± 22.96	***0.023***
Body Mass Index (BMI)	26.17 ± 4.80	23.98 ± 3.17	***0.015***
Tumor diameter(cm)	4.13 ± 2.98	2.51 ± 0.82	***0.000***
Lymph nodes metastasis [n (%)] Distant metastasis [n (%)]	58(58.62%) 8(7.69%)	13(38.26%) 2(5.88%)	***0.035*** [1.000]

The bold values were less than 0.05, showing significant difference.

### 3.2 Clinicopathological characteristics

In Tibetan-group, 94 patients were diagnosed with invasive carcinoma of no special type, accounting for 90.38%. 9 (8.65%) patients were diagnosed with invasive carcinoma of special type, including 1 with cribriform carcinoma, 2 with medullary carcinoma, 2 with papillary carcinoma, 1 with metaplastic carcinoma, 2 with malignant phyllodes tumors and 1 with mucinous carcinoma. And 1 (0.96%) patient was noninvasive carcinoma. In Han-group, 31 patients were diagnosed with invasive carcinoma of no special type, accounting for 91.18%. And 3 (8.82%) patients were diagnosed with invasive carcinoma of special type, including 2 with mucinous carcinoma and 1 with papillary carcinoma. Among Tibetan-group, 66 (65.35%) cases were ER-positive, 35(34.65%) cases were ER-negative, and 3 were unevaluated, while 25 (73.53%) cases were ER-positive, and 9(26.47%) cases were ER-negative in Han-group. As to ER status, no significant difference was found between two groups (p>0.05). In Tibetan-group, 54(53.47%) cases were PR-positive, 47(46.53%) cases were PR-negative, and 3 were unevaluated, while 20 (60.61%) cases were PR-positive, 13(39.39%) cases were PR-negative, and 1 was unevaluated in Han-group. No significant difference was observed between two groups (p>0.05). In Tibetan-group, 23 (24.21%) cases were HER-2-positive, 72(75.79%) cases were HER-2-negative, 3 were uncertain without being retested by FISH, 2 were uncertain after being retested by FISH, and 4 were unevaluated. In Han-group, 12 (36.36%) cases were HER-2-positive, 21(63.64%) were HER-2-negative, and 1 was uncertain after be retested by FISH. There was no significant difference in positive rates of HER-2 status between two groups (p>0.05). Among Tibetan-group, 75 (79.79%) cases were Ki-67 high, 19(20.21%) were Ki-67 low, and 10 were unevaluated. Among Han-group, 31 (91.18%) cases were Ki-67 high, and 3(8.82%) were Ki-67 low. In the term of Ki-67 expression, there was no significant difference between two groups (p>0.05) (**Table 2**).

**Table 2:** Clinicopathological characteristics in Tibetan-group and Han-group[n(%)].

Clinicopathological character	Tibetan-group	Han-group	p value
Pathological type			
Invasive carcinoma of no special type Invasive ductal carcinoma Others	94(90.38%) 69(66.35%) 10(9.62%)	31(91.18%) 24(70.59%) 3(8.82%)	0.747
ER			
Positive Negative	66(65.35%) 35(34.65%)	25(73.53%) 9(26.47%)	0.379
PR			
Positive Negative	54(53.47%) 47(46.53%)	20(60.61%) 13(39.39%)	0.474
Her-2			
Positive Negative	23(24.21%) 72(75.79%)	12(36.36%) 21(63.64%)	0.177
Ki67			
High Low	75(79.79%) 19(20.21%)	31(91.18%) 3(8.82%)	0.186

### 3.3 Features of molecular subtypes

Herein, cases from both groups were classified into four molecular subtypes, including Luminal A, Luminal B, HER2-enriched, and Triple negative. Among Tibetan-group, 18 (18.18%) cases were Luminal A, 46 (46.46%) cases were Luminal B, 9 (9.09%) cases were HER2-enriched, and 26 (26.26%) cases were Triple negative. In Han-group, 3 (8.82%) cases were Luminal A, 22 (64.71%) cases were Luminal B, 7 (20.59%) cases were HER2-enriched, and 2 (5.88%) cases were Triple negative. The constituent ratios of the four molecular subtypes were significantly different between Tibetan-group and Han-group (p<0.05) (**Table 3**).

**Table 3:** Molecular subtypes in Tibetan-group and Han-group [n (%)].

Group	Luminal A	Luminal B	HER2-enriched	Triple negative	Total
Tibetan-group	18(18.18%)	46(46.46%)	9(9.09%)	26(26.26%)	99
Han-group	3(8.82%)	22(64.71%)	7(20.59%)	2(5.88%)	34
Total	21	68	16	28	133

## 4 Discussion

Breast cancer is the most common cancer in women worldwide with ever-increasing incidence, and high-altitude regions are no exception (2, 9–11). Previous evidence suggested that high-altitude populations were at higher risk for breast cancer, compared with low-altitude populations. And mounting evidence presented various differences of gene expression during breast cancer process between high- and low-altitude populations (12). Compared to low-altitude regions, the most obvious distinctions of high-altitude regions are intense ultraviolet radiation, hypoxia, and low pressure. Several studies argued whether more exposure to ultraviolet ray would result in higher incidence of breast cancer. However, their conclusions were inconsistent (13, 14). Therefore, the influence of ultraviolet radiation on the development of breast cancer was still unknown. Hypoxia is closely related to tumorigenesis and tumor progression. Zhang et al. (15) induced the breast cancer stem cells by hypoxia. In their study, hypoxic tumor microenviroment greatly promoted the excessive and aberrant angiogenesis. Usually, the tumor vessels formed by angiogenesis were tortuous, dilated and excessively branched (16). Ultimately, the disorganized vasculature was inefficient for blood supply, and contributed to the hypoxic microenviroment in turn, which played a fundamental role for tumor progression (17). Previously, researchers constantly explored the pathogenic factors and gene expression of breast cancer in high-altitude regions, but few studies focused on the clinicopathological characteristics and features of molecular subtypes, which was deserved more attention and further research.

In our study, patients in Tibetan-group were all inhabitants of Tibet, which was a representative region at high altitudes. And breast cancer women in the corresponding period at low altitudes were included as controls, forming the Han-group. Our results demonstrated that the two groups had much in common, but the unique characteristics of clinical pathology and distinct features of molecular subtypes were clearly presented in Tibetan-group.

As shown in our study, the majority of patients were between 40 and 50 years. The proportion of post-menopausal patients was over 40%, and the average age at menopause ranged from 45 to 55 years. They all had no significant difference between two groups (p>0.05). But the patient delay, Body Mass Index (BMI), tumor size, and lymph node metastasis were all different with statistical significance between the two groups (p<0.05). The patient delay, defined as the period between symptoms onset and hospital visits was 7.47 ± 11.53 months in Tibetan-group, which was significantly longer than that in Han-group (7.22 ± 22.96 months) (p<0.05). To some extent, this result illustrated the fact that hospital visits of the Tibetan-group might be seriously delayed. The causes of delay were varied. Vast areas of Tibet were economically undeveloped, same as other high-altitude regions. These areas faced a relative shortage of medical resources. And some people had weak health awareness, who paid little attention to the secondary prevention for breast cancer. Besides, the long distance and steepness of the way to hospitals hindered them to seek immediate medical attention, due to the remote location of their settlements. According to the standard deviations of both figures, the patient delay fluctuated widely. However, the reasons of two groups were different. Tibet covered large areas, and districts of Tibet were far apart. There were great distinctions in economic development. In addition, the district distribution of medical resources was greatly uneven. All the reasons mentioned above were attributed to the wide fluctuation in Tibetan-group. But in Han-group, it was probably associated with the small sample size. Women in Tibetan-group were significantly fatter than those in Han-group. As with our results, previous studies found that overweight and obesity were associated not only with a higher risk of developing breast cancer, particularly in postmenopausal women, but also with worse prognosis for women of all ages (18). Compared with Han-group, tumors in Tibetan-group were significantly larger, and patients with positive lymph nodes in Tibetan-group were significantly more, suggesting that patients in Tibetan-group might have more advanced stages, which might result in worse disease outcome. We calculated the OR related to T stage to identify if exposure to altitude could influence breast cancer by ordinal logistic regression analysis. In our results, the OR was 2.45, 95% CI was 1.10-5.44. It demonstrated that exposure to high altitudes might result in more advanced T stage. Michaelson JS et al. revealed that tumor size was associated with increased lethality, such that each millimeter of tumor diameter was associated with an additional approximately 1% chance of death (19). Zheng S et al. reported that breast cancer in China showed more invasive ductal carcinoma with larger tumor size, later stage than those in the Western (p<0.001), and their results indicated that invasive breast cancer of Chinese might be more aggressive than those of the Western population (20). Risk of getting breast cancer was related to living at a higher altitude, as well as an increased risk of death (OR:1.067; p=0.030) (21). Several previous studies had reported that the lymph node status, the number of positive lymph nodes and the sites of positive lymph nodes were all important prognostic indicators of breast cancer (22, 23).

According to our results, the major pathological type was invasive carcinoma of no special type in both groups. Among them, invasive ductal carcinoma accounted for the largest proportion. In two groups, the positive rates of ER were 65.35% and 73.53%, respectively. And the positive rates of PR were 53.47% and 60.61%, respectively. They were all found with no significant difference (p>0.05). Nuclear ER and PR, also known as members of steroid receptor superfamily, were both essential molecules, coordinately contributing to the development of lobular alveolar epithelial structures of the normal mamma during puberty, menstrual cycle and pregnancy (24). ER positive cancers were reported to be well differentiated, less aggressive, and had a better prognosis (25). The positive rates of HER-2 in two groups were 24.21% and 36.36%, respectively (p>0.05). The human epidermal growth factor receptor (HER-2) was a transmembrane tyrosine kinase receptor, and also a proto-oncogene, involved in the proliferation and differentiation of mammary cells. Over-expression of HER-2 allowed mammary cells to proliferate, survive, differentiate through a signal transduction cascade regulated by PI3k/Akt and Ras/Raf/MEK/MAPK pathways (26). Breast cancer with HER-2 over-expression constituted an aggressive type of breast cancer, which tended to grow more rapidly and were at higher risk of lymph node metastasis (27). Over expression of HER-2 was associated with poor prognosis, increased resistance to endocrine therapy and poor responding to non-anthracycline, nontaxane-containing chemotherapy (28, 29). In our two groups, cases with Ki-67 high were found in 79.79% and 91.18%, respectively, with no significant difference (p>0.05). Ki-67, also known as an excellent marker of proliferation, remained active during M, G1, S, and G2 phases of the cell cycle, and absent during G0 phase (30–33).

In 2000, Perou et al. proposed molecular classification of breast cancer into intrinsic subtypes, which differed in intrinsic biology, prognosis, and response to therapy (34). It was confirmed by accumulating evidence. In 2013, the St. Gallen international breast cancer conference expert panel refined and re-iterated the value of clinicopathological surrogate definitions resembling intrinsic subtypes to guide selection of systemic adjuvant therapies (7). Ultimately, the criteria of molecular subtypes were established. In addition, molecular subtypes had been confirmed to be the prognostic predictor of breast cancer (35, 36). In previous studies, the distribution of molecular subtypes varied, which was affected by many factors, such as sample size, testing technology, ethnicity, living conditions, economy and so on (37). In our study, Luminal B was the most common subtype both in Tibetan-group and Han-group, found in 46.46% and 64.71%, respectively. Following it, triple negative (26.26%) came to be the second in Tibetan-group, whereas it was HER2-enriched (20.59%) in Han-group. Then Luminal A (18.18%) followed, and HER2-enriched (9.09%) was the least in Tibetan-group. In Han-group, it was followed by Luminal A (8.82%) and triple negative (5.88%). The distribution of subtypes was quite significantly different between two groups (p<0.05), according to the comparison of constituent ratios of the four molecular subtypes in two groups.

There are over 140 million people who live at high altitudes worldwide. Tibetans are among them. Tibetans reside in the Qinghai-Tibetan Plateau for centuries. Long-term exposure to this kind of extreme environment brings changes in breast cancer. Our study retrospectively reviewed breast cancer patients from high-altitude regions and low-altitude regions between May 2013 and March 2022 in our hospital. We analyzed the clinicopathological characteristics and features of molecular subtypes of breast cancer at high altitudes. We also compared them with breast cancer at low altitudes. We had found that breast cancer patients at high altitudes showed significantly different in the patient delay, BMI, tumor size, lymph node metastasis and the distribution of subtypes. Our results verified the unique traits of breast cancer at altitudes, which might influence treatment strategies in the future. We will keep following these cases, collecting prognostic data, and observing long-term outcomes. Based on the present study, we recommend taking steps to raise cancer awareness, guide healthy weight maintenance and breast self-examination, improve cancer screening rate, and optimize medical resource allocation. In our study, the sample size of the control group was limited, and it might bring selection bias. We reduced its influence by rigorous statistical methods. Nevertheless, our study had still broadened the understanding of this heterogenous disease and also provided valuable evidence for cancer prevention and personalized therapeutics of breast cancer at high altitudes.

Constituent ratios of the four molecular subtypes were compared between two groups (p=0.01).

## Data availability statement

The original contributions presented in the study are included in the article/supplementary material. Further inquiries can be directed to the corresponding author.

## Ethics statement

The studies involving human participants were reviewed and approved by the ethic committee of the Hospital of Chengdu Office of People’s Government of Tibetan Autonomous Region. Written informed consent from the patients was not required to participate in this study in accordance with the national legislation and the institutional requirements.

## Author contributions

All authors participated in conceptualization, writing, review and editing the manuscript. All authors contributed to the article and approved the submitted version.
